# Effects of *Mimosa caesalpiniifolia* pre-formulation on the intestinal barrier during sodium dextran sulfate-induced colitis in Wistar rats

**DOI:** 10.7705/biomedica.6611

**Published:** 2023-06-30

**Authors:** Aline Garnevi-Fávero, Karina Nascimento-da Silva, Willian Rodrigues-Ribeiro, Caroline Marcantonio-Ferreira, Patrícia Sartorelli, Leonardo Cardili, Rita de Cássia-Sinigaglia, Joice Naiara Bertaglia-Pereira, Marcelo Aparecido-da Silva, Wagner Vilegas, Marcelo José Dias-Silva, Ana Paula Ribeiro-Paiotti

**Affiliations:** 1 Laboratory of Hepatology Molecular Applied, Discipline of Gastroenterology, Universidade Federal de São Paulo, São Paulo, Brazil Universidade Federal de São Paulo Universidade Federal de São Paulo São Paulo Brazil; 2 Institute of Environmental, Chemistry and Pharmaceutical Sciences, Department of Pharmaceutics Sciences, Universidade Federal de São Paulo, Diadema, Brazil Universidade Federal de São Paulo Universidade Federal de São Paulo Diadema Brazil; 3 Laboratory of Experimental and Molecular Pathology, Department of Pathology, Universidade Federal de São Paulo, São Paulo, Brazil Universidade Federal de São Paulo Universidade Federal de São Paulo São Paulo Brazil; 4 Electron Microscopy Centre, Universidade Federal de São Paulo, São Paulo, Brazil Universidade Federal de São Paulo Universidade Federal de São Paulo São Paulo Brazil; 5 Butantan Institute, São Paulo, Brazil Butantan Institute São Paulo Brazil; 6 Pharmacognosy Laboratory, Universidade Federal de Alfenas, Alfenas, Minas Gerais, Brazil Universidade Federal de Alfenas Universidade Federal de Alfenas Alfenas Minas Gerais Brazil; 7 Institute of Biosciences, São Paulo State University, São Vicente, São Paulo, Brazil Universidade de São Paulo São Paulo State University São Vicente São Paulo Brazil

**Keywords:** Mimosa, colitis, ulcerative, inflammatory bowel diseases, herbal medicine, Mimosa, colitis ulcerosa, enfermedades inflamatorias del intestino, medicina de hierbas

## Abstract

**Introduction.:**

Anti-inflammatories, immunosuppressants, and immunobiological are commonly used in the treatment of inflammatory bowel disease. However, some patients do not present an adequate response or lose effective response during the treatment. A recent study found a potential anti-inflammatory effect of the hydroalcoholic extract of *Mimosa caesalpiniifolia* on trinitrobenzene sulfonic acid-induced colitis in Wistar rats.

**Objective.:**

To evaluate the effects of *M. caesalpiniifolia* pre-formulation on the intestinal barrier using dextran sulfate sodium-induced colitis model.

**Materials and methods.:**

Leaf extracts were prepared in 70% ethanol and dried with a Buchi B19 Mini-spray dryer using 20% Aerosil® solution. Thirty-two male Wistar rats were randomized into four groups: basal control, untreated colitis, pre-formulation control (125 mg/kg/day), and colitis treated with pre-formulation (125 mg/kg/day). Clinical activity index was recorded daily and all rats were euthanized on the ninth day. Colon fragments were fixed and processed for histological and ultrastructural analyses. Stool samples were collected and processed for analysis of the short-chain fatty acid.

**Results.:**

Treatment with the pre-formulation decreased the clinical activity (bloody diarrhea), inflammatory infiltrate, and the ulcers. Pre-formulation did not repair the epithelial barrier and there were no significant differences in the goblet cells index. There was a significant difference in butyrate levels in the rats treated with the pre-formulation.

**Conclusions.:**

The pre-formulation minimized the clinical symptoms of colitis and intestinal inflammation, but did not minimize damage to the intestinal barrier.

## Resumen

**Introducción.:**

Los antiinflamatorios, inmunosupresores e inmunobiológicos se utilizan comúnmente para tratar la enfermedad intestinal inflamatoria. Sin embargo, algunos pacientes no presentan una respuesta adecuada o pierden respuesta efectiva durante el tratamiento. En un estudio reciente, se encontró un potencial efecto antiinflamatorio del extracto hidroalcohólico de *Mimosa caesalpiniifolia* en la colitis inducida por el ácido trinitrobenceno sulfónico utilizando ratas Wistar.

**Objetivo.:**

Evaluar los efectos de la preformulación de *M. caesalpiniifolia* sobre la barrera intestinal durante la colitis inducida por sulfato de dextrano sódico.

**Materiales y métodos.:**

Los extractos de hojas se prepararon con una solución que contenía 70 % de etanol y se secaron con un secador por aspersión Mini B19 de Buchi usando una solución con 20 % de Aerosil®. Treinta y dos ratas Wistar macho se aleatorizaron en cuatro grupos: control basal, colitis sin tratar, control con preformulación (125 mg/kg/ día) y colitis tratada con preformulación (125 mg/kg/día). El índice de actividad clínica se registró diariamente y todas las ratas se sacrificaron el noveno día. Los fragmentos de colon se fijaron y se procesaron para análisis histológicos y ultraestructurales. Se recolectaron muestras de heces y se procesaron para el análisis de ácidos grasos de cadena corta.

**Resultados.:**

El tratamiento con la preformulación disminuyó la actividad clínica (diarrea sanguinolenta), el infiltrado inflamatorio y las úlceras. La preformulación no reparó la barrera epitelial y no hubo diferencias significativas en el índice de células caliciformes. Se obtuvo una diferencia significativa en los niveles de butirato en las ratas tratadas con la preformulación.

**Conclusiones:**

La preformulación minimizó los síntomas clínicos de colitis e inflamación intestinal pero no minimizó el daño a la barrera intestinal.

Inflammatory bowel diseases are characterized by chronic inflammation. Crohn’s disease and ulcerative colitis are the most prevalent forms of this group of diseases. The etiology of inflammatory bowel diseases is not well understood, but it is known that environmental, genetic and immune response factors are involved in the development of the inflammatory response ^1^^-^^3^.

In one of the hypotheses raised, the dysregulated mucosal barrier would facilitate the access of luminal antigens through the epithelium. This would induce the recruitment of lymphocytes and macrophages with the release of cytokines and several inflammatory mediators causing intestinal damage ^4^. To promote symptomatic remission, antiinflammatories, immunosuppressants, and immunobiological are commonly used in the treatment of inflammatory bowel disease ^5^. However, some patients do not present an adequate response or loss of effective response during the treatment ^5^^-^^7^. For this reason, the interest in plants with medicinal properties is extensively investigated.

*Mimosa caesalpiniifolia* (Fabaceae: Mimosoideae) is a medicinal plant popularly known as “sansão-do-campo” or “sabiá” ^8^ in the northeastern region of Brazil. The leaves and the bark were prepared as an infusion, decoction or “bottled” against hypertension, inflammatory diseases and infections ^9^^,^^10^*. Mimosa caesalpiniifolia* hydroalcoholic extract also exhibited antimicrobial, antioxidant ^11^ and cytotoxic activity against the human breast cancer cell line MCF7 ^12^ and reduced DNA damage in blood cells from cadmium-affected mice ^13^.

A phytochemical study of a *M. caesalpiniifolia* leaf extract with antifungal activity allowed the identification of 28 compounds. Apigenin 6-C-e- boivinopyranoside, apigenin 8Ceoliopyranoside, (E)-6-(2-carboxyethenyl) apigenin, (E)8(2carboxyethenyl) apigenin and 7,5”-anhydroapigenin 6<-α- (2,6-dideoxy-5-hydroxy-arabinohexopyranoside had not been previously described ^14^^,^^15^.

In a recent study, we found a potential anti-inflammatory effect of the hydroalcoholic extract of *M. caesalpiniifolia* on trinitrobenzene sulfonic acid (TNBS)-induced colitis in Wistar rats (16). Since the incorporation of plant extracts into pharmaceutical forms can improve their solubility and bioavailability, the present study proposes to evaluate the effects of *M. caesalpiniifolia* pre-formulation on the intestinal barrier during dextran sulfate sodium (DSS)induced colitis.

In the study of Silva *et al.*, members of the Mimosoideae family were well- characterized by phytochemical diversity. Polyphenols including flavonoids and tannins are the most commonly found bioactive compounds in *M. caesalpiniifolia*. Catechins were present both in the ethyl acetate fraction and in the alcoholic extract. The hydroalcoholic extract and the ethyl acetate fraction were effective for the treatment of induced colitis ^16^.

It has been suggested that *M. caesalpiniifolia* is able to contain TNBS- induced tissue damage as a result of down-regulation of the tumor necrosis factor alpha (TNF-α) expression when using low doses of *Mimosa*^16^.

In that same study, *M. caesalpiniifolia* demonstrated the ability to inhibit cyclooxygenase-2 (COX-2) expression when administered at a dose of 250 mg after colitis induction and 50 mg of the ethyl acetate fraction. Therefore, COX-2 inhibition was only achieved when *M. caesalpiniifolia* was administered at higher doses. It appears that TNF-α and COX-2 play a key role during chronic colitis caused by TNBS ^16^.

In summary, the results suggest that *M. caesalpiniifolia* attenuated colonic lesions as a result of reduced inflammation ^16^. The main difference between the previous study and the present one, in addition to the model, is that in this case a pre-formulation was made with the vegetal extract of the plant.

## Materials and methods

### Ethics approval

The study was approved by the Commission for the Use of Animals in Research (CEUA 4362180119) of UNIFESP-EPM.

### Plant material

To prepare the preformulation, the leaves of *M. caesalpiniifolia* were collected in January 2012, in the city of Alfenas, Minas Gerais, Brazil. The geographic coordinates of the collection site are 21° 24' 44.1" S, 45° 55' 19.9" W. The plant was authenticated by Dr. Geraldo Alves da Silva at the Federal University of Alfenas (UNIFAL-mG). A voucher sample (695) was deposited in the Herbarium of UNIFAL-MG.

### Pre-formulation of Mimosa caesalpiniifolia plant extract

To create the pre-formulation for rat colitis treatment, dried and powdered leaves (256 g) of *M. caesalpiniifolia* were extracted individually by simple percolation. The ethanolic extract (4 g) was dissolved in distilled water (300 ml) and then, subjected to the liquid-liquid extraction process (partition) for seven days. After extraction, the solvent was removed by a rotary evaporator and lyophilized.

*Mimosa caesalpiniifolia* lyophilized extracts were prepared in a solution with 70% ethanol and dried with a Buchi B19 Mini-spray dryer with a 20% Aerosil® solution. The operational conditions used were followed by the Medicinal Plants and Herbal Medicines Laboratory of UNIFAL-MG ^16^.

The pre-formulation was diluted in 1.0 ml of water and given orally (gavage) to the rats, according to the experimental groups, at a dose of 125 mg/kg/day.

### Experimental design

We used 32 male Wistar rats (*Rattus norvegicus albinus*) from the Center for the Development of Experimental Models for Medicine and Biology (CEDEME) of UNIFESP-EPM. These animals weighted between 200 and 250 g and were kept in rooms with restricted access, controlled temperature (23°C) and a 12 hours-light period, 12 hours-dark cycle. Standard laboratory chow and drinking water were provided *ad libitum*. The rats were randomized into four groups: basal control (BC), 5% untreated DSS-induced colitis (DSS), MC pre-formulation control (125 mg/kg/day) (MCC), and 5% DSS-induced colitis treated with MC pre-formulation (125 mg/kg/day) (DSSMC).

### Induction of colitis and euthanasia

A 5% DSS solution (MP Biomedicals) was administered to induce colitis, diluted in water and offered *ad libitum* for five days. Vials containing the DSS solution were changed to maintain drug efficacy on the third day. The DSS solution was changed back to drinking water after five days. The *M. caesalpiniifolia* pre-formulation was diluted in 1.0 ml of water and administered by oral gavage per six days. The clinical activity index (CAI) -percentage of weight loss, stool consistency and rectal bleeding- was recorded daily using the score ^17^.

All rats were euthanized on the ninth day with a lethal dose of halothane (Tanohalo®). Cardiac puncture was performed to confirm death ten minutes after the total loss of reflexes. Abdominal cavity was open to extract the entire colon, and then we washed it with 0.9% saline solution to remove fecal residues.

### Histopathological analysis

After dissection, the distal colon was fixed in 10% buffered formalin and soaked in paraffin. Tissue sections of 3 µm were stained with hematoxylin and eosin for histopathological analysis of colon damage by light microscopy. The images were captured at 250X magnification with an Axio cam MRc5 camera attached to an Axioskop 40 Zeiss™ optical microscope. Setting and image acquisition were performed using Axio Vision software. The scale bar was inserted with the same software ^18^.

### Transmission electron microscopy

To analyze colon damage, samples from rat’s colon (two from each group) measuring 1 mm χ 1 mm χ 2 mm were fixed in 2% formaldehyde plus 2.5% glutaraldehyde in 0.1 M sodium cacodylate buffer (pH 7.2) for two hours at 4°C. Subsequently, samples were fixed in 2% osmium tetroxide in 0.1 M sodium cacodylate buffer for the same period. The samples were washed in phosphate buffered saline solution (PBS), dehydrated with ethanol, soaked in mounting medium at 37 °C for three hours and finally polymerized with EPON epoxy resin at 60 °C for 36 hours.

We used semithin sections (300-500 µm) for area delimitation. The slides were subjected to light microscopy and hot stained with 1% toluidine blue solution. Subsequent analyzes were carried out using a transmission electron microscope, model EX II 1200 (JEOL, Japan).

### Goblet cells index

For mucin analysis of the goblet cells, the material was stained using alcian blue histochemical technique and counterstained with hematoxylin for identification and quantification of goblet cells.

The alcian blue dye (Ventana) was added to the sections for 20 minutes and rinsed in distilled water. Hematoxylin was applied to the sections for 30 seconds, followed by rinsing in running water, alcohol and xylene, and mounted in Entellan resin (Merck).

About 15 fields were captured in five different sections for each animal, containing approximately 1,000 consecutive cells. Indistinctly of alcian blue positivity, we quantified the cells with an Olympus BX 40™ optical microscope at 200X magnification. The ratio between the number of goblet cells and the number of enterocytes in the mucosa was defined as the goblet cell index (CI), as follows: CI = (Number of goblet cells / total number of mucous cells) x 100.

### Short-chain fatty acids measurement

To indicate the level of intestinal epithelial barrier protection we measured short-chain fatty acids according to the protocol of Ribeiro *et al.*^19^ with adaptations.

To quantify short-chain fatty acids, we generate a standard curve for the concentration range of 0.15610 mmol/L using rat feces as a matrix.

Fecal samples were weighed into microtubes (approximately 30 mg per sample) and homogenized with 100 µl of distilled water. Then, we added 40 mg of sodium chloride, 50 µl of 1M hydrochloric acid and 300 µl of n-butanol. The microtubes were vortexed for two minutes and centrifuged at 18,000*g* for 15 minutes. The organic phase was transferred to chromatographic vials and analyzed using a GC-FID 2010 (Shimadzu) equipped with an AOC20i Autosampler and fused silica capillary wax columns (Rtx) of 30 mm x 0.25 mm (Restek Corp.) coated with 0.25 µm polyethylene glycol. Samples (1 µl) were injected at 260 °C using a 20:1 split ratio. High-grade pure nitrogen was used as carried gas at 1 ml/minute constant flow and the results were expressed in terms of concentration (μΜ).

### Statistical analysis

We performed one-way analysis of variance (ANOVA), followed by Tukey’s post-hoc test using Graph Pad Prism (version 6.0). The significance level adopted was p<0.05 (α = 5%). Data were recorded as mean plus or minus standard deviation (SD): mean ± SD.

## Results

### Effects of Mimosa caesalpiniifolia pre-formulation on the clinical activity index

**Figure 1:**
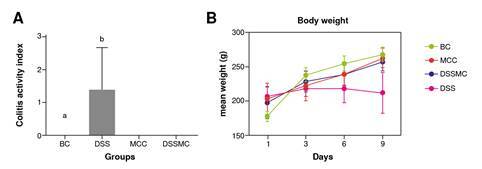
Effects of *Mimosa caesalpiniifolia* pre-formulation on the clinical activity index. A) Total clinical score activity index: ^a^ basal control vs 5% untreated dextran sulphate sodium (DSS) induced colitis - p=0.0003; ^b^ 5% untreated DSS-induced colitis (DSS) vs *Mimosa caesalpiniifolia* pre formulation control and 5% DSS-induced colitis treated with *Mimosa caesalpiniifolia* preformulation (DSSMC) - p=0.0003; B) Body weight. There were non-significant diferences between groups according to Tukey's test (eight rats per group).

BC: basal control; DSS: dextran sulphate sodium; MCC: *Mimosa caesalpiniifolia* pre formulation control; DSSMC: DSS-induced colitis treated with *Mimosa caesalpiniifolia* pre-formulation

As expected, the animals in the basal control group remained healthy from the beginning to the end of the experiment, showing gain weight. The only administration of *M. caesalpiniifolia* preformulation in the control group did not cause diarrhea or weight loss, demonstrating therapeutic safety. On the other hand, the animals in the untreated DSS-induced colitis group showed all the clinical signs such as weight loss, diarrhea, and rectal bleeding. Treatment with the *M. caesalpiniifolia* pre-formulation attenuated colitis symptoms (p=0.0003). The results of this analysis are summarized in figure 1.

**Figure 2:**
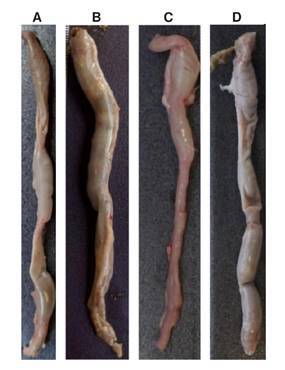
Images of Wistar rat’s intestine in each of the four groups. **A)** Basal control - without macro-scopic changes; **B)** 5% untreated DSS-induced colitis presented intestinal thickening, edema, and ulcers; **C)** *Mimosa caesalpiniifolia* pre-formulation control (125 mg/kg/ day) did not present macroscopic chang-es; and **D)** 5% DSS-induced colitis treated with *Mimosa caesalpiniifolia* pre-formulation (125 mg/kg/day) presented mild edema and hyperemia

During the macroscopic evaluation, the rats of the basal control and *M. caesalpiniifolia* control showed total integrity of the colon, without signs of edema, inflammation, or ulcers. In contrast, the untreated DSS-induced colitis group showed thickening, edema, and ulcers. Rats with DSS-induced colitis treated with *M. caesalpiniifolia* pre-formulation showed mild edema and hyperemia. The macroscopic representation of the colon from each group can be viewed in figure 2.

### Effects of *Mimosa caesalpiniifolia* pre-formulation on the microscopic score

**Figure 3:**
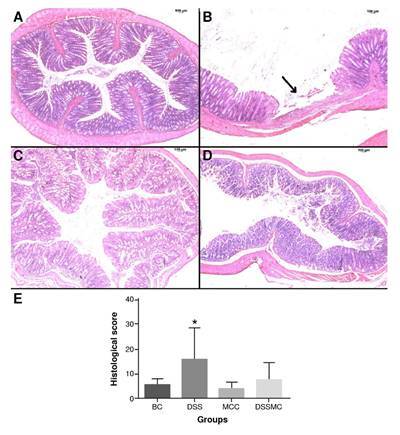
Effects of *Mimosa caesalpiniifolia* pre-formulation on intestinal tissue stained with hematoxilin-eosin, 250X. **A)** Basal control with typical cellular architecture; **B)** 5% untreated DSS-induced colitis showed intense inflammation and presence of ulcer (arrow) alternated by healthy areas; **C)** *Mimosa caesalpiniifolia* pre-formulation control with typical cellular architecture; **D)** 5% DSS-induced colitis treated with *Mimosa caesalpiniifolia* pre-formulation with typical cellu-lar architecture; and **E)** Histological score.

BC: basal control; DSS: dextran sulphate sodium; MCC: *Mimosa caesalpiniifolia* pre formulation control; DSSMC: DSS-induced colitis treated with *Mimosa caesalpiniifolia* pre-formulation

There were no changes in the colonic cellular architecture of the basal and *M. caesalpiniifolia* groups. In the DSS-induced colitis group, we observed intense inflammatory exudate and the presence of ulcers, permeated by healthy areas. On the other hand, rats treated with *M. caesalpiniifolia* preformulation showed a decrease in histological score. There was a reduction of the inflammatory infiltrate cells and the absence of ulcers, but no significant differences. The representative photomicrographs of each group and the histological score are in figure 3.

There were significant differences (p=0.0187) between * 5% untreated DSS-induced colitis and *Mimosa caesalpiniifolia* pre-formulation control according to Tukey's test (eight rats per group).

### Effects of Mimosa caesalpiniifolia pre-formulation on goblet cell's index

**Figure 4:**
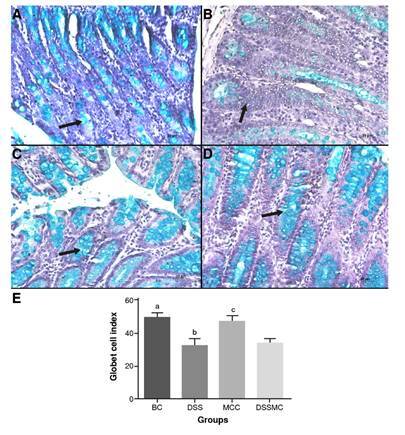
Effects of *Mimosa caesaipiniifolia* pre-formulation on goblet cell's index. Samples stained with Alcian blue staining, 500X. **A)** Basal control with preserved goblet cells (arrow); **B)** 5% untreated DSS induced colitis showed depletion of goblet cells (arrow); **C)** *Mimosa caesaipiniifolia* pre-formulation control with preserved goblet cells (arrow); **D)** 5% DSS-induced colitis treated with *Mimosa caesaipiniifoiia* preformulation with preserved goblet cells; and **E)** Goblet cells index.

BC: basal control; DSS: dextran sulphate sodium; MCC: Mimosa caesalpiniifolia pre formulation control; DSSMC: DSS-induced colitis treated with Mimosa caesalpiniifolia pre-formulation

There were no significant differences in the goblet cells index between the group treated with *M. caesalpiniifolia* pre-formulation when compared with the DSS-induced colitis group. The results are summarized in figure 4.

Statistically significant differences (p<0.0001) were found comparing BC versus DSS and DSSMC; DSS versus MCC; MCC versus DSSMC. Analyses were performed with five rats per group applying ANOVA and Tukey's post-hoc test.

### Effects of Mimosa caesalpiniifolia pre-formulation on ultrastructural changes

**Figure 5:**
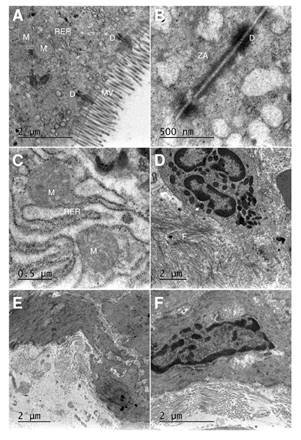
Representative transmission electron micrographs: Basal control displaying typical morphology of mucosal villus: zonula adherens preserved, normal rugous endoplasmic reticulum, preserved desmosome, and mitochondria. **A)** 2 µm scale, and **B)** 0.5 µm scale; 5% untreated DSS-induced colitis displaying edema of rugous endoplasmic reticulum, swollen mitochondria, a deposit of collagen fiber, loss of mucosal villus and zonula adherens. **C)** 0.5 µm scale and **D)** 2 µm scale; **E)** and **F)** 5% DSS induced colitis treated with *Mimosa caesaipiniifolia* pre-formulation displaying a deposit of collagen fiber, loss of mucosal villus and zonula adherens, at a 2 µm scale.

MV: mucosal villus; ZA: zonula adherens; RER: rugous endoplasmic reticulum; D: desmosome; M: mito-chondria; F: collagen fiber

The basal control group showed preserved organelles, absorptive epithelium, and tight junctions. The DSS-induced colitis group displayed disorganization of the mitochondrial crest, endoplasmic reticulum edema, nucleus fragmentation, collagen fiber deposit (healing process indicative), junctions, and absorptive epithelium loss. We observed that the treatment with *M. caesalpiniifolia* pre-formulation stalled the inflammatory process, but was not able to restore the absorptive epithelium and the tight junctions. The ultrastructural changes can be viewed in figure 5.

### Effects of Mimosa caesalpiniifolia pre-formulation on SCFAs measurement

**Figure 6:**
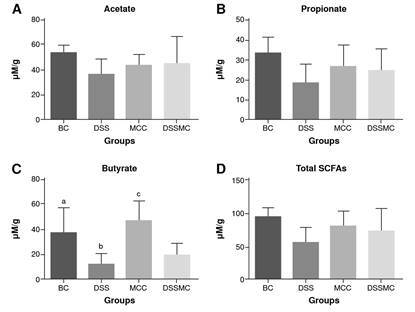
Effects of *Mimosa caesalpiniifolia* pre-formulation on short-chain fatty acids (SCFAs) measurements. Non-significant differences were found in SCFAs for acetate (A), propionate (B) and total SCFAs levels (D) in each of the four assessed groups

BC: basal control; DSS: dextran sulphate sodium; MCC: *Mimosa caesalpiniifolia* pre formulation control; DSSMC: DSS-induced colitis treated with *Mimosa caesalpiniifolia* pre-formulation

The retention time for the short-chain fatty acids was 7.53 minutes for acetic acid, 8.28 for propionic acid, and 9.18 for butyric acid. The total run time was 13 minutes. Overall, the results show that the levels of short-chain fatty acids are decreased in rats of the DSS-induced colitis group, mainly for butyrate levels (p=0.0040). Although no significant differences were evident, we observed that treatment with *M. caesaipiniifoiia* pre-formulation demonstrated a trendy increase of short-chain fatty acids (figure 6).

Statistically significant differences (p=0.0040) were found for butyrate levels (C) comparing: BC versus 5% untreated DSS-induced colitis; DSS versus *Mimosa caesalpiniifolia* pre-formulation control, and MCC versus 5% DSS-induced colitis treated with *Mimosa caesalpiniifolia* preformulation (DSSMC). Analyses were per-formed with six rats per group applying ANOVA and Tukey's post-hoc test.

## Discussion

Researchers are highly attracted to using plants in folk medicine, as this is ancient knowledge. Bioactive compounds such as flavonoids and catechin derivatives that have antioxidant, anti-inflammatory, and healing actions, among others, are revealed through phytochemical screening of different species ^20^.

Other species of *Mimosa* have also been applied to treat other diseases ^21^. *Mimosa pudica*, for example*,* is used in the treatment of various activities and has shown anthelmintic action ^22^ and anti-inflammatory properties ^23^. The *M. pudica* root extract contains high levels of antioxidants ^24^.

*Mimosa tenuiflora* is a popular remedy used in Mexico and Brazil. The bark of this plant is an effective remedy for treating burns and wounds, and is also used to prevent inflammation ^23^^-^^26^.

Another species, the *Mimosa hostilis,* also known as jurema, is culturally used in Candomblé rituals of indigenous heritage ^27^. The ingestion of jurema wine, or the consumption of the stem and root in the form of smoke, triggers psychotropic effects such as euphoria, visual and oneiric hallucinations, and perception distortion of time, space, shape and colors, among other symptoms. In addition, *M. hostilis* is also used against coughs and it aids in wound healing ^27^.

In a previous study, we found that administration of hydroalcoholic extract of *M. caesalpiniifolia* at a dose of 125 mg/kg after induction of colitis by TNBS was able to mitigate the harmful effects by decreasing colon inflammatory process through negative regulation of TNF-α expression ^16^^,^^28^. With these findings and to evolve in the development of a plantbased medication, the extract of *M. caesalpiniifolia* was incorporated into the adjuvant Aerosil®, obtaining the pre-formulation at a promising dose of 125 mg/kg. We tested the preformulation as a possible restorer of the intestinal barrier in rats with DSS-induced colitis.

It is well-established that during the clinical activity of inflammatory bowel diseases in humans, there is disruption of the intestinal barrier caused by the intense inflammatory response triggered, powering the process of intestinal injury ^4^^,^^29^. In this context, the DSSinduced colitis is the most assertive way to evaluate the mechanisms of intestinal barrier injury ^30^^-^^32^. Initially, the disruption of the colon epithelial monolayer allows the dissemination of pro- inflammatory stimuli (microorganisms and their secreting proteins) into the underlying tissue ^30^^-^^32^. Indeed, in our study we successfully induced colitis with bloody diarrhea and weight loss as main symptoms. We emphasized the therapeutic safety of the *M. caesalpiniifolia* pre-formulation, since the control group did not show intestinal alterations. The rats with DSSinduced colitis and treated with the pre-formulation demonstrated a significant decrease in the clinical signs of colitis, eliminating episodes of diarrhea and promoting gradual weight gain.

In the clinical management of inflammatory bowel diseases, the goal is remission of the clinical symptoms and complete healing of the intestinal mucosa. In this point of view, the *M. caesalpiniifolia* pre-formulation might be a good option due to its healing and antiinflammatory properties. Although there were no significant differences, we observed decreasing histological damage, stalling of the inflammatory process, and a massive deposit of collagen fiber in animals treated with *M. caesalpiniifolia* pre-formulation. However, there was no evidence of barrier restoration. Like the untreated DSS-induced colitis group, there was a loss of absorptive epithelium and tight junctions, endoplasmic reticulum stress, and disorganization of the mitochondrial crest.

These findings corroborate the mechanisms of injury in classical human inflammatory bowel diseases, where disturbances in the intestinal barrier lead to mitochondrial damage, alteration in the ATP production causing reactive oxygen species (ROS) accumulation, hindering cellular metabolism, and macromolecules anabolism for effective repair ^33^^-^^38^. Also, the endoplasmic reticulum stress promotes ROS increasing, which leads to the goblet cells’ death, affecting the processes of synthesis, modification and folding of proteins, particularly those of the mucin 2 protein (MUC-2), secreted continuously to replenish the mucus layer ^39^. Based on this fact, we also evaluated the effects of *M. caesalpiniifolia* pre-formulation in the goblet cells index, but there was no significant differences. Therefore, our findings suggested that the treatment could prevent the death of goblet cells by minimizing the damage caused by DSS.

Butyrate, acetate, and propionate are considered energy sources for colonocytes, stimulating smooth muscle contractions, intestinal blood flow, transepithelial chloride secretion chloride, and exerting stimuli of colonic epithelial cells ^40^^,^^41^. In addition, some studies reported that low concentrations of butyrate stimulate *MUC2* expression suggesting a protective effect on intestinal barrier function ^40^^,^^41^. Although no significant differences were observed, our findings of the short-chain fatty acid measurement suggest that *M. caesalpiniifolia* preformulation treatment positively affected the levels of acetate, propionate, and butyrate.

The *M. caesalpiniifolia* pre-formulation minimized the clinical symptoms of colitis and intestinal inflammation but did not minimize damage to the intestinal barrier. We hypothesized that a new experimental protocol with a prolonged treatment period could demonstrate more effective results regarding the role of *M. caesalpiniifolia* pre-formulation on epithelial barrier restoration.

Therefore, we can conclude that the treatment with the pre-formulation of the *M. caesalpiniifolia* plant extract in the experimental colitis induced by the DSS reduced the clinical symptoms when administered alone, and helped reduce microscopic lesions. On the other hand, it did not restore the damage to the intestinal barrier and did not modify the index of goblet cells, nor interfere significantly with the levels of short-chain fatty acids. In addition, we suggest that the DSS model may demonstrate a better reproducibility of UC in the animals compared to the TNBS model, which would be better for a colitis disease model.

Phytotherapy requires more in-depth studies and care by qualified professionals, remembering that it is not because it is natural that it is not harmful. Popular medicine in Brazil has not finished its course, it is in constant formation and transformation, incorporating new elements at every moment.
